# Efficacy and Safety of Regenerative Periodontal Therapy on Recovery After Surgical Removal of Impacted Third Molars: A Systematic Review and Meta‐Analysis

**DOI:** 10.1111/odi.70150

**Published:** 2025-12-15

**Authors:** Xueping Ou, Shaofei Ma

**Affiliations:** ^1^ Department of Stomatology, Minghang Hospital Fudan University Shanghai China; ^2^ Department of Pathology, Shanghai General Hospital Shanghai Jiaotong University Shanghai China

**Keywords:** impacted third molars, meta‐analysis, recovery, regenerative periodontal therapy, systematic review

## Abstract

**Objective:**

To assess the efficacy and safety of regenerative periodontal therapy (RPT) in recovery after impacted third molar extraction.

**Methods:**

A systematic search was conducted in PubMed, Embase, Web of Science, and the Cochrane Library to identify eligible randomized controlled trials (RCTs) published from database inception to January 31, 2025. Pooled analyses were performed using a random‐effects model. Effect estimates were reported as weighted mean difference (WMD) for continuous outcomes and odds ratio (OR) for categorical outcomes, both with 95% confidence intervals (CI).

**Results:**

Thirty‐two RCTs involving 1300 participants were included. Compared with conventional treatment, RPT significantly improved clinical outcomes, including greater clinical attachment level gain (WMD: 1.69; 95% CI: 1.13–2.25; *p* < 0.001), probing depth reduction (WMD: 1.13; 95% CI: 0.48–1.79; *p* = 0.001), and alveolar bone level gain (WMD: 1.31; 95% CI: 0.62–2.01; *p* < 0.001). The intervention demonstrated a comparable safety profile to the control group, with no statistically significant difference in adverse event risk (OR: 0.64; 95% CI: 0.34–1.19; *p* = 0.154). Subgroup analyses revealed significant heterogeneity in efficacy and safety across different regenerative protocols.

**Conclusion:**

Current evidence indicates that RPT enhances periodontal tissue regeneration after third molar extraction, leading to improved clinical outcomes without increasing adverse events.

**Trial Registration:**

INPLASY: INPLASY202540109

## 1 Introduction

The third molar, as the last permanent tooth to erupt in the human oral cavity, is the most frequently impacted tooth due to its unique anatomical position (Lima et al. 2012). Modern anthropological evidence attributes this high impaction rate to an evolutionary mismatch: while jaw size has reduced over time, tooth dimensions have remained stable (Passi et al. 2019). Key contributing factors include insufficient jaw development (limiting eruption space), ectopic eruption of adjacent teeth, abnormal bone density creating mechanical barriers, and discrepancies between dental arch length and tooth mass (Passi et al. 2019). Epidemiological studies reveal wide variations in third molar impaction prevalence across populations (9.5%–68%), with over half of individuals exhibiting at least one impacted third molar—most commonly in the mandible (Passi et al. 2019). Mesioangular impaction predominates among impaction types, followed by horizontal and vertical orientations (Passi et al. 2019). Impacted third molars may lead to significant pathological consequences: (1) recurrent pericoronitis from food retention; (2) a 3–5‐fold increased risk of caries on the distal surface of adjacent teeth (Syed et al. 2017); and (3) secondary damage such as root resorption and periodontal tissue destruction—almost exclusively limited to the second molar adjacent to the extracted third molar (Hashemipour et al. 2013). Notably, the prognosis of impacted third molars correlates strongly with second molar health. When the contact point lies below the cementoenamel junction, adjacent surface caries incidence reaches 54%—significantly higher than in noncontact scenarios (Syed et al. 2017). These findings highlight the critical need for early evaluation and intervention.

Mandibular third molar extraction remains the most common oral surgical procedure, yet poses technical challenges due to anatomical complexity and procedural risks (Vranckx et al. 2022; Barone et al. 2021; Antonelli et al. 2023). Within 72 h postoperatively, 78.2% of patients experience pain, 65.4% facial swelling, and 53.8% develop limited mouth opening. These symptoms typically persist for 5–7 days, impairing essential functions like eating and speech (Bailey et al. 2020). Furthermore, approximately 42.7% of extractions cause distal periodontal damage to adjacent teeth, with bony impactions showing a 58.3% incidence rate (Pardo et al. 2023). Common manifestations include increased probing depth (PD) ≥ 4 mm, ≥ 2 mm clinical attachment loss (CAL), and elevated bleeding on probing (Pardo et al. 2023). Such complications underscore the importance of immediate postoperative periodontal assessment and early intervention.

Critically, regenerative periodontal therapy (RPT) is uniquely positioned to address these post‐extraction challenges by targeting the root causes of poor recovery: (1) It mitigates adjacent tooth periodontal damage by promoting CAL gain—directly reversing attachment loss and reducing PD, which are the primary drivers of long‐term second molar pathology (Syed et al. 2017; Pardo et al. 2023); (2) It offsets alveolar bone resorption by stimulating new bone formation, preserving ridge volume for future restorative care; and (3) It may reduce postoperative morbidity by accelerating soft tissue healing—addressing the functional impairments that affect patient quality of life (Bailey et al. 2020). Unlike conventional treatment, RPT proactively regenerates lost tooth‐supporting structures rather than merely managing symptoms, representing a significant therapeutic advancement for patients at high risk of post‐extraction complications.

RPT aims to functionally restore tooth‐supporting structures lost to periodontal disease or trauma. The gold standard achieves: (1) three‐dimensional regeneration of the periodontal ligament with properly oriented Sharpey fibers; (2) new bone that biologically integrates with the periodontal interface; and (3) bioactive cementum covering the root surface (Polimeni et al. 2006). Current clinical protocols combine bioactive agents, optimized barrier membranes for guided tissue regeneration, precision bone grafting, and three‐dimensional space maintenance strategies (Camps‐Font et al. 2018). While RPT significantly improves CAL and promotes bone formation, histological evidence indicates true cementum regeneration occurs in only 23%–41% of cases, with reparative cementum being more prevalent (Camps‐Font et al. 2018). Notably, existing evidence on RPT for post‐impacted third molar extraction recovery remains limited and fragmented: (1) prior systematic reviews and meta‐analyses either focused on a single RPT modality or had small sample sizes, failing to comprehensively compare the efficacy of diverse RPT techniques (Bao et al. 2021; Vitenson et al. 2022; Ramos et al. 2022; Ye et al. 2024); (2) no previous study has systematically analyzed technique‐specific safety profiles—critical for clinical decision‐making—such as whether osseous grafting increases adverse events or PRP reduces complications; (3) heterogeneity in RPT protocols has not been adequately explored, hindering the translation of evidence to personalized clinical practice. Given these findings—and to ensure robust causal inferences about RPT's therapeutic impact—we conducted a systematic review and meta‐analysis of randomized controlled trials (RCTs) to evaluate RPT's efficacy and safety in post‐extraction recovery of impacted third molars.

## 2 Materials and Methods

### 2.1 Data Sources, Search Strategy, and Selection Criteria

This systematic review and meta‐analysis were conducted in accordance with the Preferred Reporting Items for Systematic Reviews and Meta‐Analyses (PRISMA) guidelines (Page et al. 2021), comparing the efficacy of RPT with standard treatment or spontaneous healing following impacted third molar extraction. Our study was registered in the INPLASY platform (number: INPLASY202540109). A comprehensive search was performed across PubMed, Embase, Web of Science, and the Cochrane Library through January 31, 2025. All eligible RCTs were included in this study regardless of publication language or status (study was formally published in a peer‐reviewed journal or remained unpublished/unpeer‐reviewed). Specifically, our search for ‘unpublished/unpeer‐reviewed’ studies was targeted at identifying completed trial results from clinical trial registries and conference abstracts that presented sufficient raw data for extraction. Master's or PhD theses were not included in this review, as our protocol specifically focused on the study design (RCTs) rather than the publication format, and theses often lack the stringent peer‐review and standardized reporting of interventional RCTs.

The search strategy adopted a combined approach of compound medical subject headings: (“third molars”) AND (“regeneration” OR “wound healing” OR “guided tissue regeneration, periodontal” OR “bone substitutes”) AND “randomized controlled trial”. The detailed search formula is available in File S1. Additional searches were conducted for completed but unpublished studies via the ClinicalTrials.gov registry (National Institutes of Health, NIH, USA). A snowballing method was also applied by manually screening reference lists from included articles and relevant systematic reviews to identify additional eligible studies.

Two reviewers independently conducted literature searches and study selection. Discrepancies were resolved through consensus, with a third reviewer consulted when necessary. The inclusion criteria were as follows: (1) participants: systemically healthy patients requiring impacted third molar extraction; (2) intervention: RPT delivered to anatomical sites directly affected by third molar extraction—specifically: (a) the distal surface of the second molar adjacent to the extracted third molar (for modalities targeting periodontal defect repair: guided tissue regeneration [GTR], platelet‐rich concentrate [PRP]); or (b) the extraction socket of the extracted third molar (for modalities targeting bone preservation/augmentation: guided bone regeneration [GBR], osseous grafting); or similar site‐specific regenerative techniques; (3) comparator: either standard treatment or spontaneous healing. “Standard treatment” specifically included: (a) gentle irrigation of the extraction socket with sterile saline to remove debris; (b) primary soft tissue closure with absorbable sutures to protect the socket; (c) postoperative instructions; (d) prescription of oral analgesics for pain management; and (e) selective use of prophylactic antibiotics for 3–5 days—with no use of RPT modalities; (4) outcomes: clinical outcomes measured at the distal surface of the second molar adjacent to the extracted third molar (the primary site of post‐extraction periodontal damage (Pardo et al. 2023)): CAL gain, PD reduction, and alveolar bone level (ABL) gain; plus incidence of adverse events at the extraction site; and (5) study type: limited to RCTs. Our primary objective—evaluating the causal efficacy and safety of RPT versus conventional treatment—requires minimizing bias that is inherent to observational designs and could distort estimates of treatment effect. Specifically: (1) observational studies often enroll participants nonrandomly, leading to confounding by indication; (2) key factors affecting post‐extraction recovery are often incompletely recorded in observational data. Without randomization to balance these factors between RPT and control groups, residual confounding could obscure true treatment effects; and (3) observational studies can only demonstrate associations, not causation—critical for a clinical question where we aim to recommend RPT as an evidence‐based intervention. RCTs, by contrast, use randomization to distribute known and unknown confounders evenly across groups, enabling more robust causal inferences about RPT's impact on CAL, PD, ABL, and adverse events. While observational studies can provide valuable real‐world data (e.g., long‐term RPT utilization patterns), they are not suited to answering our core question of “whether RPT causes improved post‐extraction recovery.” Restricting to RCTs aligns with the Cochrane Handbook's guidance for systematic reviews evaluating therapeutic interventions, where RCTs are prioritized to minimize bias and maximize the reliability of effect estimates.

### 2.2 Data Collection and Quality Assessment

A structured data extraction framework was used to systematically collect the following research characteristics: (1) Basic information: surname of the first author, publication year, research region, and trial design type; (2) Demographic characteristics: total sample size, baseline age distribution, and sex composition; (3) Technical parameters: surgical site positioning criteria, regenerative treatment protocol, and control measures; and (4) Efficacy indicators: primary and secondary clinical outcomes and detailed reports of adverse events.

Methodological quality was assessed using the Cochrane Collaboration's Risk of Bias Assessment Tool, with quantitative evaluation based on the following domains: random sequence generation, allocation concealment, participant and personnel blinding, outcome assessment blinding, incomplete outcome data, selective reporting, and other bias (Higgins et al. 2022). Data extraction and quality assessment were conducted independently by two reviewers. Discrepancies were resolved through a three‐stage consensus process (independent review → cross‐validation → adjudication by senior researchers), with all adjudications referencing the full‐text records of the original studies.

### 2.3 Statistical Analysis

Effect size indicators were selected based on variable type: for continuous efficacy outcomes of RPT, the weighted mean difference (WMD) with its 95% confidence interval (95% CI) was used; for dichotomous adverse event data, the odds ratio (OR) and 95% CI were calculated. The DerSimonian–Laird random‐effects model was applied to estimate pooled effects. This model adjusts weight distribution by incorporating between‐study variance (*τ* ^2^), helping correct for heterogeneity bias arising from differences in study design and participant characteristics (DerSimonian and Laird 1986; Ades et al. 2005). A two‐dimensional heterogeneity assessment was performed. The *I* ^*2*^ statistic quantified heterogeneity, and Cochran's *Q* test was used to test its significance. Per Cochrane Handbook guidelines, significant heterogeneity was defined as meeting both of the following: (1) *I* ^*2*^ ≥ 50%; and (2) *Q* test *p* value < 0.10 (Deeks et al. 2008; Higgins et al. 2003). Sensitivity analysis was conducted using the leave‐one‐out method. This involved sequentially removing each study, recalculating the pooled effect size and 95% CI, comparing results with the original model, and evaluating the impact of individual studies. Unstable results were identified when any of the following occurred: effect direction reversed; effect size changed by ≥ 20%; or statistical significance crossed the threshold. All analyses confirmed result stability (Tobias 1999). Subgroup analyses examined RPT effects by intervention type. Differences among subgroups were evaluated using interaction tests, assuming normal distribution of the data (Altman and Bland 2003). The GRADE (Grading of Recommendations, Assessment, Development, and Evaluations) approach (Guyatt et al. 2008) was used to methodically assess the certainty of evidence, evaluating five key areas: risk of bias, inconsistency, indirectness, imprecision, and publication bias. The overall certainty ratings spanned from high to very low. Publication bias was assessed through funnel plot inspection and quantitatively tested using Egger's linear regression and Begg's rank correlation tests. When funnel plot asymmetry was evident and either test yielded a *p* value < 0.10, publication bias was considered present (Egger et al. 1997; Begg and Mazumdar 1994). All *p* values for pooled estimates were two‐sided, with *p* < 0.05 considered statistically significant. Analyses were performed using STATA (version 12.0; StataCorp, College Station, Texas, USA).

## 3 Results

### 3.1 Literature Search

**FIGURE 1:**
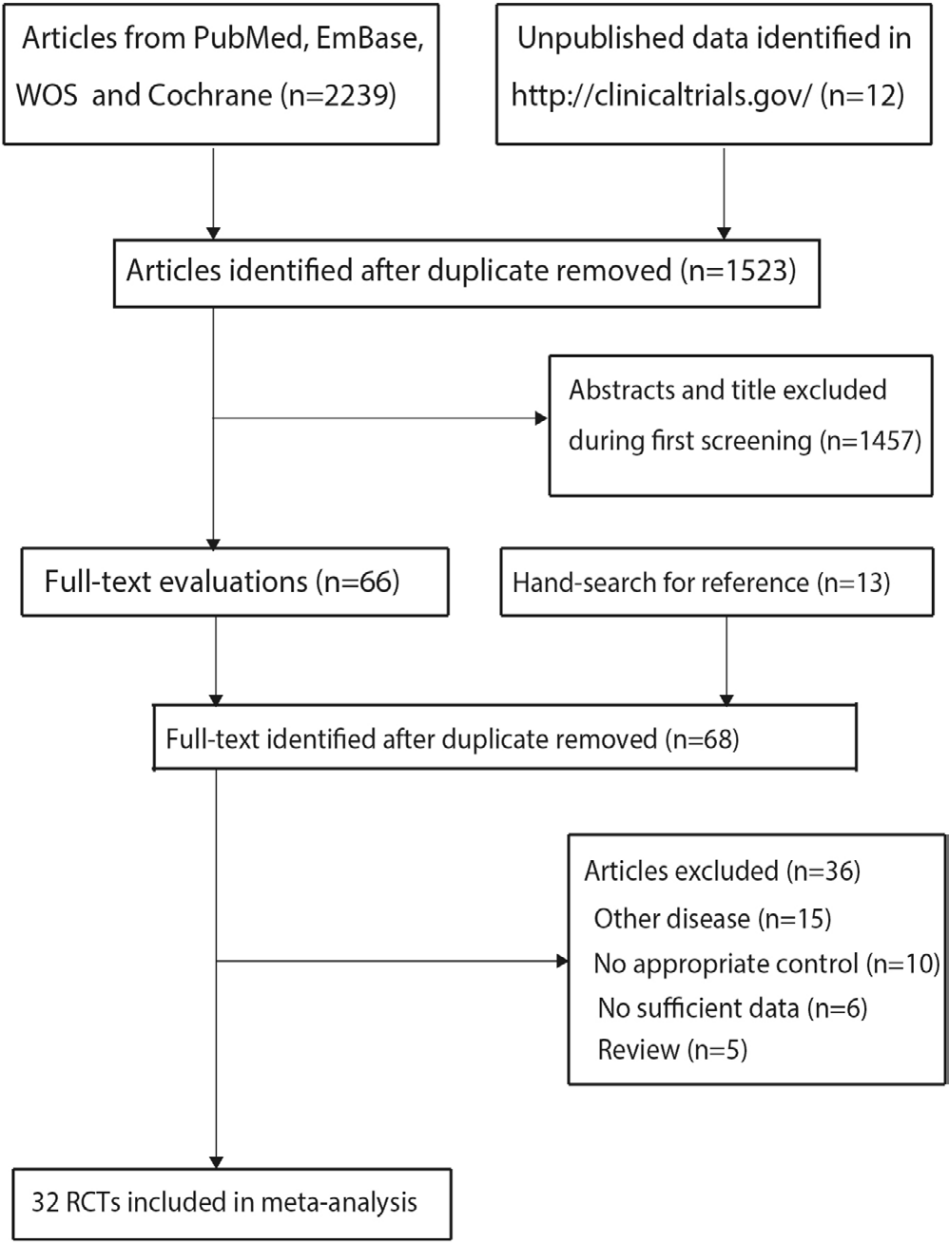
Flow diagram of the literature search and study selection process.

A total of 2239 records were initially identified through electronic databases. After removing duplicates, 1523 unique records remained. Following the screening of titles and abstracts, 1457 were excluded for not meeting the inclusion criteria. The full texts of the remaining 66 records were assessed, and 34 were excluded due to inconsistent disease status (*n* = 15)—defined as deviation from our inclusion criterion requiring “systemically healthy patients undergoing extraction of impacted third molars,” absence of an effective control group (*n* = 8), insufficient data (*n* = 6), or being review articles (*n* = 5) (File S2). Manual searching identified 13 additional records, 11 of which overlapped with the electronic search and 2 of which were excluded for lacking appropriate controls. Ultimately, 32 RCTs were included in the meta‐analysis (Figure 1) (Pecora et al. 1993; T. B. Dodson 1996, 2004; Oxford et al. 1997; Karapataki et al. 2000; Throndson and Sexton 2002; Sammartino et al. 2005, 2009; Aimetti et al. 2007; Andrade Munhoz et al. 2011; Ruga et al. 2011; Hassan et al. 2012; Eshghpour et al. 2014; Cortell‐Ballester et al. 2015; Kumar et al. 2015; Singh et al. 2015; Kilinc and Ataol 2017; Ge et al. 2017; Asutay et al. 2017; Daugela et al. 2018; Unsal et al. 2018; Zahid and Nadershah 2019; Kim et al. 2020; Gasparro et al. 2020; Özveri Koyuncu et al. 2020; Elayah et al. 2022; O'Sullivan et al. 2022; Fang et al. 2022; Sun et al. 2023; Iqbal et al. 2023; Moraes et al. 2024; Zwittnig et al. 2024).

### 3.2 Study Characteristics

This meta‐analysis included 32 clinical studies. Of these, 24 (75.0%) used a split‐mouth design, and 8 (25.0%) were parallel‐controlled trials. As shown in Table 1, the studies involved a total of 1300 participants, with sample sizes ranging from 10 to 118. Geographically, 22 studies (68.8%) were conducted in Western countries (or regions), and 10 (31.2%) in Eastern countries (or regions). Methodological quality, assessed using a standardized tool, was generally moderate to high (Table 2).

**TABLE 1:** The baseline characteristics of included studies and involved participants.

Study	Country	Study design	Sample size	Age (years)	Male (%)	Surgical site	Intervention	Control
Pecora et al. (1993)	Brazil	Parallel groups	18	26.0–50.0	NA	Horizontally inclined, impacted mandibular 3M and ≥ 5 mm periodontal pocket and pathosis distally to 2M	E‐PTFE membrane	Spontaneous healing
T. B. Dodson (1996)	USA	Split‐mouth	14	21.7	28.6%	Bilateral impacted 3Ms	Allograft	Spontaneous healing
Oxford et al. (1997)	USA	Split‐mouth	12	21.1	NA	Bilateral, mesioangularly inclined, soft tissue‐impacted 3 Ms	E‐PTFE membrane	Spontaneous healing
Karapataki et al. (2000)	Sweden	Split‐mouth	20	35.2	NA	Bilateral, mesioangularly or horizontally inclined, impacted 3Ms. and 4 mm ODD distally to 2M	PLA resorbable membrane	Spontaneous healing
Throndson and Sexton (2002)	USA	Split‐mouth	14	31.0	35.7	Bilateral and completely soft tissue‐impacted 3Ms	Bioactive glass particles	Spontaneous healing
T. B. Dodson (2004)	USA	Split‐mouth	12	31.3	62.5	Bilateral and impacted 3Ms	Allograft +Collagen resorbable membrane; Collagen resorbable membrane	Spontaneous healing
Sammartino et al. (2005)	Italy	Split‐mouth	18	21.0–26.0	55.6	Bilateral, mesioangularly inclined, fully impacted 3Ms and preoperative PD ≥ 7.5 mm and CAL ≥ 6 mm distally to 2M	Platelet‐rich plasma	Spontaneous healing
Aimetti et al. (2007)	Italy	Split‐mouth	15	24.9	NA	Bilateral, mesioangularly inclined, completely soft tissue‐impacted 3Ms. and ≥ 4 mm periodontal pocket and BoP distally to 2M	PGA/PLA resorbable membrane	Spontaneous healing
Sammartino et al. (2009)	Italy	Split‐mouth	45	21.0–30.0	55.6	Bilateral, mesioangularly or horizontally inclined, fully impacted 3Ms. and preoperative PD ≥ 7 mm and CAL ≥ 6 mm distally to 2M	Xenograft + Collagen resorbable membrane; Collagen resorbable membrane	Spontaneous healing
Andrade Munhoz et al. (2011)	Brazil	Split‐mouth	22	15.0–25.0	45.5	Symmetric impacted 3Ms	Composite xenograft + Bovine resorbable membrane	Spontaneous healing
Ruga et al. (2011)	Italy	Split‐mouth	14	29.5	42.9	Bilateral, mesioangularly inclined, and completely bony or soft tissue impacted 3Ms	Platelet‐rich fibrin	Spontaneous healing
Hassan et al. (2012)	Egypt	Split‐mouth	14	32.0	57.1	Bilateral, horizontally inclined, impacted 3Ms	Xenograft + Collagen resorbable membrane	Spontaneous healing
Eshghpour et al. (2014)	Iran	Split‐mouth	78	25.1	42.3	Symmetric impacted 3Ms	Platelet‐rich fibrin	Spontaneous healing
Cortell‐Ballester et al. (2015)	Spain	Parallel groups	56	34.7	51.8	Mesioangularly or horizontally inclined and completely soft tissue impacted 3M	Collagen resorbable membrane	Spontaneous healing
Kumar et al. (2015)	India	Parallel groups	31	26.1	NA	Mesioangularly or horizontally inclined, impacted 3M	Platelet‐rich fibrin	Spontaneous healing
Singh et al. (2015)	India	Split‐mouth	25	25.4	52.0	Bilateral impacted 3Ms	Hydroxyapatite with collagen	Absorbable gelatin sponge
Kilinc and Ataol (2017)	Turkey	Parallel groups	90	23.1	26.7	Mesioangularly or vertical, inclined, partially impacted 3Ms	Collagen resorbable membrane	Spontaneous healing
Ge et al. (2017)	China	Split‐mouth	51	27.5	46.7	Bony‐impacted 3M and preoperative ODD ≥ 4 mm distally to 2M	Particulate autograft	Spontaneous healing
Asutay et al. (2017)	Turkey	Split‐mouth	30	20.3	20.0	Bilateral symmetric impacted third molars	Platelet‐rich fibrin	Spontaneous healing
Daugela et al. (2018)	Lithuania	Split‐mouth	34	22.8	41.2	Bilateral extractions of impacted 3Ms	Leukocyte‐and platelet‐rich fibrin	Regular blood clot control
Unsal et al. (2018)	Turkey	Split‐mouth	50	24.0	34.0	Bilateral symmetric partially erupted mandibular 3Ms	Platelet‐rich fibrin	Spontaneous healing
Zahid and Nadershah (2019)	Saudi Arabia	Split‐mouth	10	24.0	0.0	Bilateral impacted 3Ms	Advanced platelet‐rich fibrin	Spontaneous healing
Kim et al. (2020)	Korea	Split‐mouth	31	23.5	48.4	Bilateral mandibular impacted 3 Ms	Absorbable collagen sponge	Spontaneous healing
Gasparro et al. (2020)	Italy	Split‐mouth	18	23.3	55.6	Clinical attachment loss on the distal site to the 2Ms. associated with impacted 3Ms. in both sides of the jaw	Leukocyte‐and platelet‐rich fibrin	Spontaneous healing
Özveri Koyuncu et al. (2020)	Turkey	Split‐mouth	60	25.8	35.0	Impacted mandibular 3Ms	Concentrated growth factor	Spontaneous healing
Elayah et al. (2022)	China	Split‐mouth	37	25.0	51.4	Bilaterally and symmetrically impacted lower 3 Ms	Concentrated growth factor	Spontaneous healing
O'Sullivan et al. (2022)	Ireland	Parallel groups	74	28.1	23.0	Unilateral impacted 3Ms	Plasma rich in growth factors	Spontaneous healing
Fang et al. (2022)	China	Parallel groups	118	30.0	55.9	Mesioangular, horizontal, vertical, partially impacted 3 Ms	Concentrated growth factor fibrin	Spontaneous healing
Sun et al. (2023)	China	Parallel groups	66	29.3	47.0	The impacted mandibular 3 Ms. were showed mesioangular or horizontal	Autogenous bone, Bio‐Oss with barrier membrane; concentrated growth factor gel with concentrated growth factor membrane	Regular blood clot control
Iqbal et al. (2023)	Pakistan	Parallel groups	170	24.2	51.2	Unilateral mandibular 3 Ms	Platelet‐rich fibrin	Spontaneous healing
Moraes et al. (2024)	Brazil	Split‐mouth	28	22.4	42.9	Bilateral impacted 3Ms	Leukocyte‐and platelet‐rich fibrin	Spontaneous healing
Zwittnig et al. (2024)	Austria	Split‐mouth	25	≥ 16.0	24.0	Bilateral impacted 3Ms	Platelet‐rich fibrin	Spontaneous healing

**TABLE 2:** The methodological quality assessment of included trials.

Study	Random sequence generation	Allocation concealment	Blinding of participants and personnel	Blinding of outcome assessment	Incomplete outcome data	Selective reporting	Other bias
Pecora et al. (1993)	Low	Low	High	Unclear	Low	Low	High
T. B. Dodson (1996)	High	Low	Low	High	High	Low	Unclear
Oxford et al. (1997)	Unclear	Low	High	Unclear	Low	Low	High
Karapataki et al. (2000)	Low	Low	High	High	Low	Low	Unclear
Throndson and Sexton (2002)	Unclear	Low	Unclear	Unclear	High	Low	High
T. B. Dodson (2004)	Low	Low	Low	High	Low	Low	Unclear
Sammartino et al. (2005)	Low	Low	Low	Low	Low	Low	Low
Aimetti et al. (2007)	Low	Low	Low	Low	Low	Low	Unclear
Sammartino et al. (2009)	Low	Low	Low	Low	Low	Low	Low
Andrade Munhoz et al. (2011)	Low	Low	High	Low	High	Low	High
Ruga et al. (2011)	Low	Low	High	High	Low	Low	Unclear
Hassan et al. (2012)	Low	Low	Low	Low	Low	Low	Low
Eshghpour et al. (2014)	Low	Low	Low	Low	Low	Low	Low
Cortell‐Ballester et al. (2015)	Low	Low	Low	Low	Low	Low	Low
Kumar et al. (2015)	Low	Low	High	Low	Low	Low	Unclear
Singh et al. (2015)	Unclear	Low	Low	Low	Low	Low	Unclear
Kilinc and Ataol (2017)	Low	Low	High	Low	Low	Low	Unclear
Ge et al. (2017)	Low	Low	High	Low	Low	Low	Unclear
Asutay et al. (2017)	Low	Low	Unclear	Low	Low	Low	Unclear
Daugela et al. (2018)	Low	Low	Low	Low	Low	Low	Unclear
Unsal et al. (2018)	Low	Low	Low	Low	Low	Low	Low
Zahid and Nadershah (2019)	Low	Low	Low	Low	Low	Low	Unclear
Kim et al. (2020)	Low	Low	Low	Low	Unclear	Low	Unclear
Gasparro et al. (2020)	Low	Low	Low	Low	Low	Low	Low
Özveri Koyuncu et al. (2020)	Low	Low	Low	Low	Low	Low	Low
Elayah et al. (2022)	Low	Low	Low	Low	Low	Low	Unclear
O'Sullivan et al. (2022)	Low	Low	Low	Low	Low	Low	Low
Fang et al. (2022)	Low	Low	Low	Low	Low	Low	Low
Sun et al. (2023)	Low	Low	Unclear	Low	Low	Low	Low
Iqbal et al. (2023)	Low	Low	Low	Low	Low	Low	Low
Moraes et al. (2024)	Low	Low	Low	Low	Low	Low	Unclear
Zwittnig et al. (2024)	Low	Low	Low	Low	Low	Low	Low

### 3.3 CAL Gain

**FIGURE 2:**
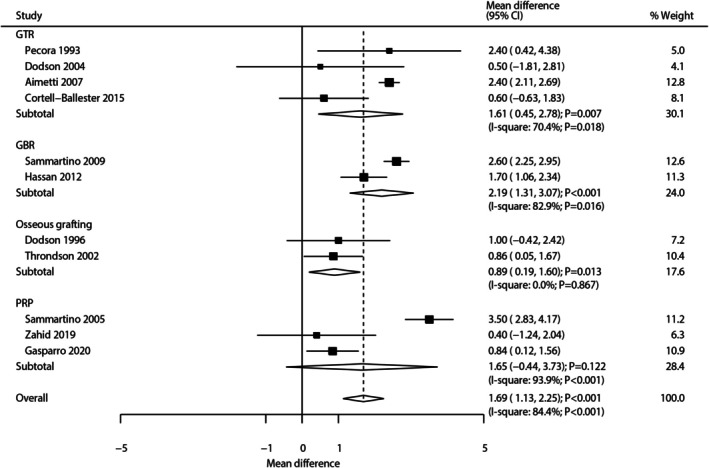
Effect of regenerative periodontal therapy on clinical attachment level gain.

Eleven trials reported the effect of RPT on CAL gain. Importantly, all trials measured CAL at the distal surface of the second molar adjacent to the extracted third molar—the site of most significant post‐extraction periodontal attachment loss. The pooled result showed that RPT was associated with significantly greater CAL gain (WMD: 1.69; 95% CI: 1.13–2.25; *p* < 0.001), with substantial heterogeneity across studies (*I* ^*2*^ = 84.4%; *p* < 0.001) (Figure 2). This 1.69 mm CAL gain exceeds the threshold of ≥ 1 mm that defines as “clinically relevant” for periodontal therapy. In clinical practice, this means RPT helps preserve long‐term function of the second molar—a common site of post‐extraction periodontal damage. Sensitivity analysis confirmed the robustness of the results upon sequential removal of individual trials (File S3). Subgroup analyses indicated significant CAL gains with GTR (WMD: 1.61; 95% CI: 0.45–2.78; *p* = 0.007), GBR (WMD: 2.19; 95% CI: 1.31–3.07; *p* < 0.001), and osseous grafting (WMD: 0.89; 95% CI: 0.19–1.60; *p* = 0.013), while PRP showed no significant effect (WMD: 1.65; 95% CI: −0.44 to 3.73; *p* = 0.122) (Table 3). Potential publication bias was observed (Egger's test: *p* = 0.059; Begg's test: *p* = 0.755; File S4).

**TABLE 3:** Summary of subgroup analysis outcomes by regenerative periodontal therapy modality.

Outcome measure	RPT modality	No of studies	WMD/OR and 95% CI	*p*	*I* ^2^ (%)	*Q* statistic
Clinical attachment level gain	GTR	4	1.61 (0.45 to 2.78)	0.007	70.4	0.018
	GBR	2	2.19 (1.31 to 3.07)	< 0.001	82.9	0.016
	Osseous grafting	2	0.89 (0.19 to 1.60)	0.013	0.0	0.867
	PRP	3	1.65 (−0.44 to 3.73)	0.122	93.9	< 0.001
Probing depth reduction	GTR	7	1.22 (0.18 to 2.26)	0.021	96.6	< 0.001
	GBR	1	1.50 (1.02 to 1.98)	< 0.001	—	—
	PRP	7	0.99 (−0.01 to 1.99)	0.052	93.0	< 0.001
Alveolar bone level gain	GTR	5	1.13 (0.21 to 2.06)	0.017	86.9	< 0.001
	GBR	1	2.40 (2.17 to 2.63)	< 0.001	—	—
	Osseous grafting	2	1.07 (−0.72 to 2.87)	0.241	84.3	0.012
Adverse events	GTR	11	0.92 (0.33 to 2.63)	0.882	60.7	0.882
	Osseous grafting	3	6.87 (1.13 to 41.64)	0.036	0.0	0.975
	PRP	8	0.31 (0.18 to 0.55)	< 0.001	0.0	0.666

### 3.4 PD Reduction

**FIGURE 3:**
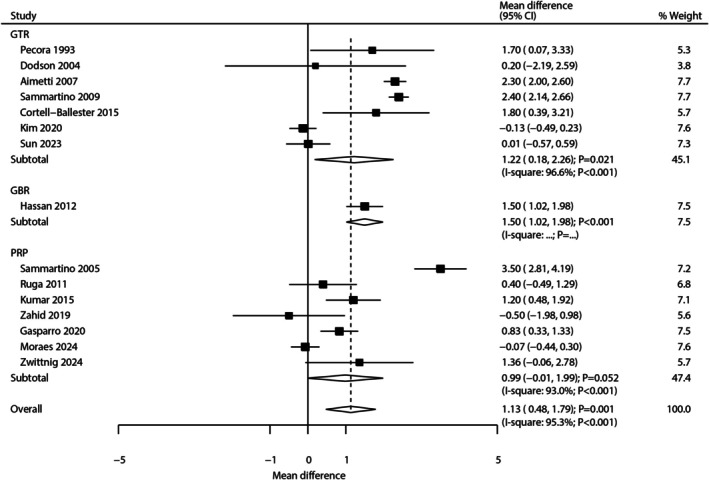
Effect of regenerative periodontal therapy on probing depth reduction.

Fifteen trials assessed the effect of RPT on PD reduction. All trials measured PD at the distal surface of the second molar adjacent to the extracted third molar—the primary site of pathological pocket formation after third molar extraction. The pooled result showed that RPT significantly increased PD reduction (WMD: 1.13; 95% CI: 0.48–1.79; *p* = 0.001). Considerable heterogeneity was observed (*I* ^*2*^ = 95.3%; *p* < 0.001) (Figure 3). This reduction moves many post‐extraction pockets from the “pathological” (≥ 4 mm) to “stable” (< 4 mm) range, reducing the need for subsequent periodontal interventions and lowering the risk of adjacent tooth caries. Sensitivity analysis confirmed the pooled conclusion remained stable following sequential exclusion of individual trials (File S3). Subgroup analyses indicated significant PD reduction with GTR (WMD: 1.22; 95% CI: 0.18–2.26; *p* = 0.021) and GBR (WMD: 1.50; 95% CI: 1.02–1.98; *p* < 0.001), whereas PRP did not significantly affect PD reduction (WMD: 0.99; 95% CI: −0.01 to 1.99; *p* = 0.052) (Table 3). For GBR, this result is based on a single study and cannot be considered statistically significant or generalizable—subgroup analyses require ≥ 3 studies to assess consistency and minimize random error. No significant publication bias was detected (Egger's test: *p* = 0.459; Begg's test: *p* = 0.843; File S4).

### 3.5 ABL Gain

**FIGURE 4:**
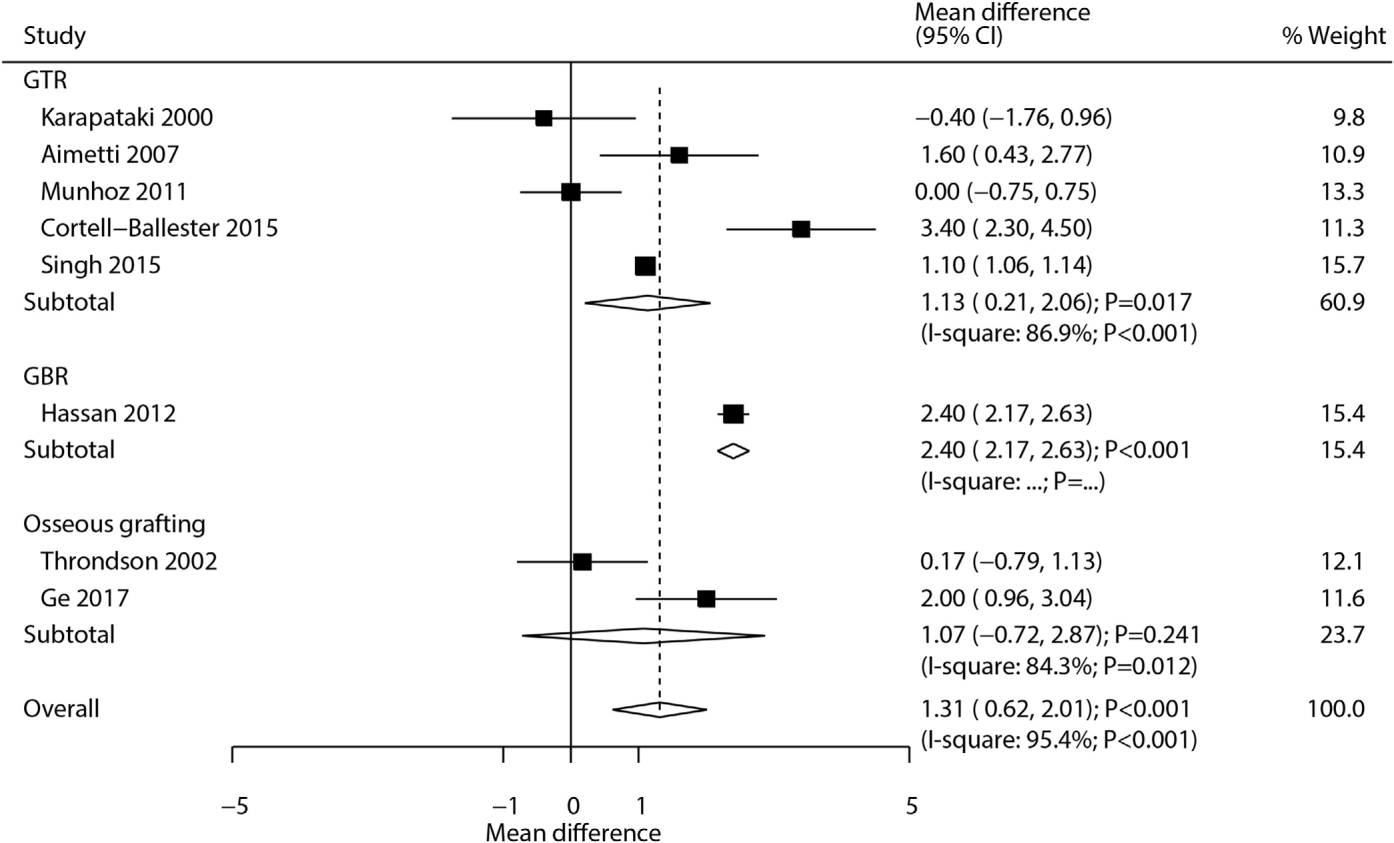
Effect of regenerative periodontal therapy on alveolar bone level gain.

Eight trials evaluated the effect of RPT on ABL gain. All trials measured ABL at the distal surface of the second molar adjacent to the extracted third molar—the site where post‐extraction alveolar bone resorption is most pronounced. Pooled analysis showed a significant increase in ABL gain (WMD: 1.31; 95% CI: 0.62–2.01; *p* < 0.001), with substantial heterogeneity (*I* ^2^ = 95.4%; *p* < 0.001) (Figure 4). This 1.31 mm ABL gain addresses a critical clinical challenge: post‐extraction alveolar bone resorption. By offsetting this resorption, RPT preserves alveolar ridge volume—essential for future dental implant placement and maintenance of facial contour. The pooled conclusion was stable and unaffected by the sequential removal of individual trials (File S3). Subgroup analyses revealed significant ABL gain with GTR (WMD: 1.13; 95% CI: 0.21–2.06; *p* = 0.017) and GBR (WMD: 2.40; 95% CI: 2.17–2.63; *p* < 0.001), whereas osseous grafting showed no significant effect (WMD: 1.07; 95% CI: −0.72 to 2.87; *p* = 0.241) (Table 3). For GBR, this finding is derived from a single study and cannot be interpreted as statistically significant—pooled analyses and significance testing are inappropriate for subgroups with < 3 studies. No evidence of publication bias was found (Egger's test: *p* = 0.526; Begg's test: *p* = 0.711; File S4).

### 3.6 Adverse Events

**FIGURE 5:**
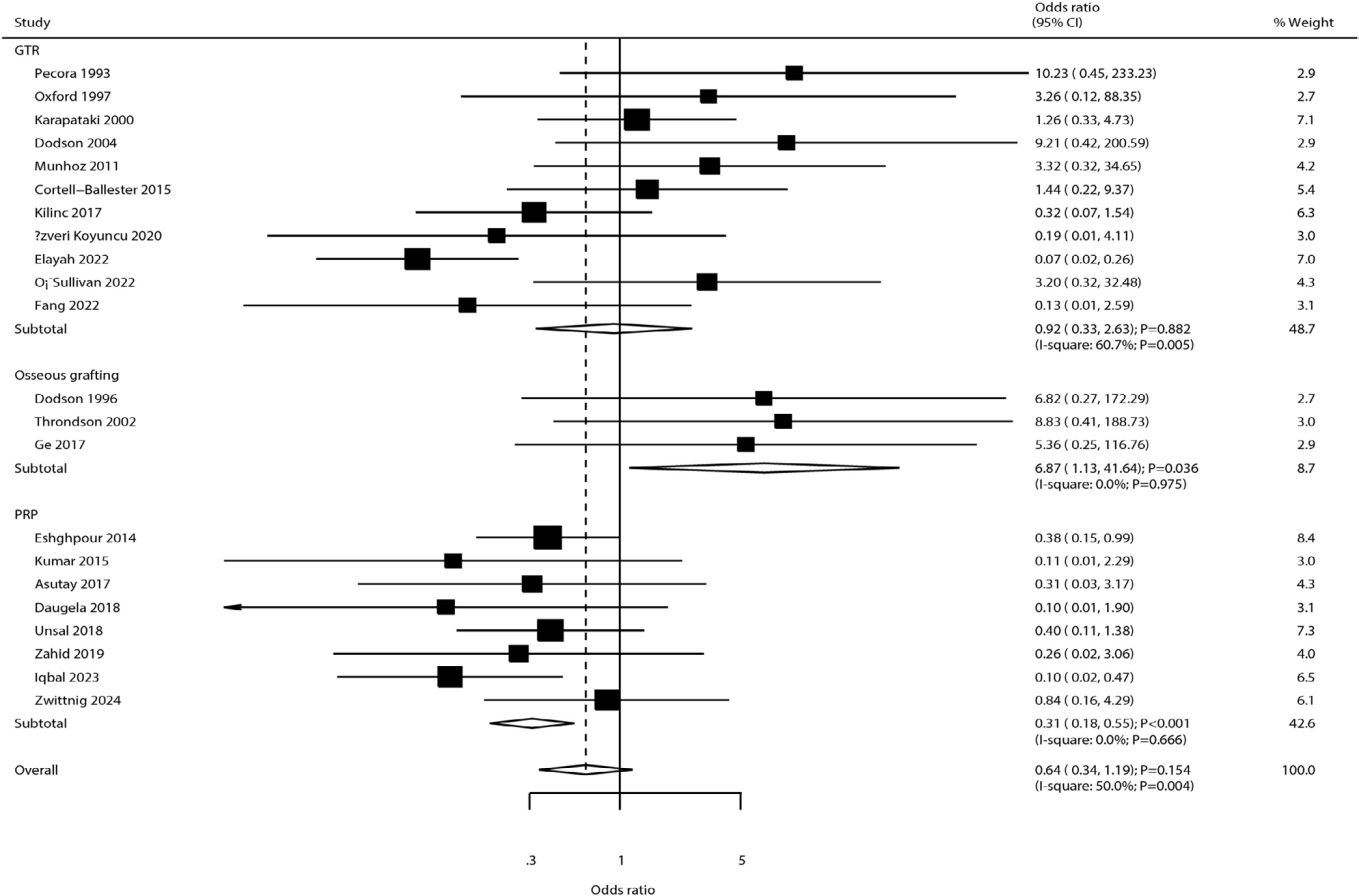
Effect of regenerative periodontal therapy on the risk of adverse events.

Twenty‐two trials reported on adverse events associated with RPT. Overall, RPT was not significantly associated with adverse event risk (OR: 0.64; 95% CI: 0.34–1.19; *p* = 0.154), although moderate heterogeneity was present (*I* ^2^ = 50.0%; *p* = 0.004) (Figure 5). Sensitivity analysis confirmed result robustness, with no changes upon removal of individual trials (File S3). Subgroup analysis indicated an increased risk with osseous grafting (OR: 6.87; 95% CI: 1.13–41.64; *p* = 0.036) and a reduced risk with PRP (OR: 0.31; 95% CI: 0.18–0.55; *p* < 0.001), while GTR was not significantly associated with adverse events (OR: 0.92; 95% CI: 0.33–2.63; *p* = 0.882) (Table 3). Significant publication bias was noted (Egger's test: *p* = 0.055; Begg's test: *p* = 0.037; File S4).

### 3.7 Evidence Quality Assessment Using the GRADE Tool

We used the GRADE tool to assess the quality of evidence for four key outcomes: CAL gain, PD reduction, ABL gain, and adverse events. The GRADE framework classifies evidence quality as high, moderate, low, or very low, starting with an initial rating (high for RCTs) and adjusting for five domains of potential limitation: risk of bias, inconsistency, indirectness, imprecision, and publication bias. Detailed Rationale for Ratings is summarized in Table 4 and listed as follows: (1) Risk of bias: no downgrade. Most included RCTs had low‐to‐moderate risk of bias; this did not significantly compromise evidence integrity; (2) Inconsistency: downgraded by 1 level for all outcomes. CAL gain, PD reduction, and ABL gain exhibited high‐to‐very high heterogeneity, though subgroup analysis partially explained variability. Adverse events had moderate heterogeneity, consistent with expected variability in surgical complication reporting; (3) Indirectness: no downgrade. All studies enrolled systemically healthy patients undergoing impacted third molar extraction, and outcomes directly matched our research question—no gaps in generalizability to clinical practice; (4) Imprecision: no downgrade. The pooled sample size and narrow 95% CIs provided sufficient precision to detect clinically meaningful effects; and (5) Publication bias: downgraded by 1 level only for adverse events.

**TABLE 4:** GRADE evidence quality summary for primary outcomes.

Outcome measure	Initial quality	Downgrading factors	Final GRADE quality
CAL gain	High	−1 (Inconsistency: high heterogeneity, partially explained by biomaterial type)	Moderate
PD reduction	High	−1 (Inconsistency: very high heterogeneity, only partially explained by biomaterial type)	Moderate
ABL gain	High	−1 (Inconsistency: very high heterogeneity, partially explained by biomaterial type)	Moderate
Adverse events	High	−1 (Inconsistency: moderate heterogeneity) + −1 (publication bias: Egger's test *p* = 0.055)	Moderate

## 4 Discussion

This large‐scale meta‐analysis of 32 RCTs, encompassing 1300 participants, comprehensively evaluated the efficacy and safety of RPT following impacted third molar extraction. The results demonstrated that RPT significantly outperformed conventional treatments across key clinical indicators: (1) CAL gain: mean improvement of 1.69 mm, with the greatest effects observed in GTR, GBR, and osseous grafting subgroups; (2) PD reduction: average decrease of 1.13 mm, particularly pronounced in the GTR subgroup; and (3) ABL gain: mean improvement of 1.31 mm. Safety assessments revealed no statistically significant difference in overall adverse event rates between RPT and control groups. However, technique‐specific heterogeneity was evident: osseous grafting significantly increased adverse events, PRP reduced complication risk, and GTR showed no significant association with adverse outcomes.

Previous systematic reviews have reported mixed efficacy for platelet‐rich fibrin (PRF) and its derivatives in third molar extraction recovery: (1) Bao et al. (2021) (10 RCTs) found that leukocyte‐rich PRF and modified PRF alleviated symptoms but did not standardize clinical outcomes; (2) Vitenson et al. (2022) (four studies) noted that advanced PRF (A‐PRF) reduced postoperative pain by 29%–42% but had limited effects on swelling and trismus; (3) Ramos et al. (2022) (11 RCTs) confirmed that leukocyte‐PRF and A‐PRF improved swelling control by 19%–24% over standard PRF but did not mitigate temporomandibular dysfunction; and (4) Ye et al. (2024) (33 trials) reported that PRF reduced dry socket incidence by 58% and enhanced bone mineral density by 21.5% over 6 months. Despite these findings, existing studies lack long‐term evaluations of RPT, particularly regarding alveolar ridge preservation and functional reconstruction. Although one prior meta‐analysis aligned with our results on overall efficacy (Camps‐Font et al. 2018), it was limited by a small sample size and did not assess technique‐specific outcomes. While we recognize that RPT for post‐extraction recovery of impacted third molars has been discussed in prior studies, our work advances the field through three key novel contributions that address unmet needs in the existing literature: (1) unprecedented scale and generalizability: most prior systematic reviews and meta‐analyses on this topic are limited by small sample sizes and narrow study inclusion. In contrast, our study synthesizes 32 RCTs involving 1300 participants—the largest sample size to date for evaluating diverse RPT techniques—and includes both Western and Eastern populations, enhancing the generalizability of our findings to global clinical practice; (2) first comprehensive technique‐specific efficacy and safety profiling: no prior study has systematically compared the efficacy (CAL gain, PD reduction, ABL gain) and safety (adverse event risk) of all four major RPT modalities (GTR, GBR, osseous grafting, PRP) in a single analysis; and (3) resolution of heterogeneity and robustness of evidence: prior meta‐analyses noted high heterogeneity in RPT efficacy but failed to explore its sources or validate result stability. Our study addresses this by conducting subgroup analyses to link heterogeneity to RPT technique type, performing rigorous leave‐one‐out sensitivity analysis, confirming that no single study biases our pooled results, and quantifying publication bias with both Egger's and Begg's tests, providing a more transparent assessment of evidence reliability than prior works.

This study demonstrated that RPT significantly improves CAL gain, PD reduction, and ABL gain. RPT integrates growth factors and biomaterials that stimulate the proliferation and differentiation of periodontal ligament stem cells, leading to extracellular matrix and collagen fiber secretion. This promotes reattachment of the periodontal ligament to the root surface, thereby enhancing CAL (Di Vito et al. 2023). Osteogenic growth factors, including bone morphogenetic proteins, induce mesenchymal stem cell differentiation into osteoblasts, which synthesize and mineralize the bone matrix. This process increases alveolar bone volume and improves ABL (Sallum et al. 2019). Furthermore, RPT incorporates anti‐inflammatory agents that reduce inflammation, thereby minimizing periodontal tissue damage from inflammatory cells. As a result, the periodontal pocket depth decreases (Chandrasekar et al. 2015). Lastly, RPT utilizes barrier membranes that inhibit migration of gingival epithelial and connective tissue cells toward the root, allowing periodontal ligament cells to occupy this space and reattach to the root surface (Hägi et al. 2014).

Further subgroup analysis revealed that GTR, GBR, and osseous grafting demonstrated significantly greater improvements in CAL gain. In contrast, PRP showed no significant effect in this regard. This is primarily because PRP, while rich in growth factors that promote cellular proliferation and differentiation, lacks the physical barrier function of membranes used in GTR and GBR. As a result, it cannot effectively guide the directional growth and attachment of cells. Additionally, without appropriate scaffold materials, PRP's growth factors may dissipate rapidly, limiting their sustained therapeutic effect and thereby diminishing its impact on CAL gain (Madi and Elakel 2021). Regarding PD reduction and ABL gain, GTR exhibited more pronounced therapeutic benefits. PRP did not significantly reduce PD. Although PRP facilitates tissue repair via the release of growth factors, it cannot reduce pocket depth effectively, as it lacks the structural capacity for guided tissue regeneration provided by GTR (Madi and Elakel 2021). Osseous grafting also showed no significant effect on ABL gain. While it can fill bone defects, poor integration between the graft material and host bone, coupled with insufficient vascularization or bioactive support, may impair bone regeneration outcomes (Camps‐Font et al. 2018).

In comparing adverse events, no significant overall difference was found between RPT and control groups. However, subgroup analysis indicated that osseous grafting increased adverse event risk, whereas PRP was associated with a reduction. Materials used in osseous grafting may provoke immune responses, and procedures such as tissue stripping and drilling can injure adjacent soft tissues, blood vessels, or nerves. Moreover, insufficient integration of grafted bone with host tissue may further contribute to complications. Conversely, PRP is abundant in growth factors such as platelet‐derived growth factor and vascular endothelial growth factor, which promote cellular proliferation, differentiation, and migration, thereby accelerating tissue repair and regeneration. PRP also contains coagulation factors released by platelets, enhancing hemostasis and reducing postoperative bleeding and hematoma formation. By facilitating rapid wound closure, PRP lowers the incidence of complications such as infection, wound dehiscence, and hematoma.

The GRADE tool provides a structured framework to translate evidence into practice, where moderate‐quality evidence (our final rating for all outcomes) supports conditional to strong recommendations—depending on the balance of benefits, harms, and patient values. Below we integrate GRADE results with our efficacy/safety findings and clinical significance data to derive actionable recommendations: (1) Recommendation for RPT in patients at risk of post‐extraction periodontal damage: Moderate‐quality evidence shows RPT improves CAL (1.69 mm), PD (1.13 mm), and ABL (1.31 mm)—with no increased overall adverse events (moderate‐quality evidence). This benefit is particularly critical for patients with pre‐existing second molar periodontal compromise, where RPT reduces future periodontal breakdown; (2) Modality‐specific recommendations: *GBR*: Strong recommendation for patients prioritizing bone/tissue regeneration. Moderate‐quality evidence shows GBR achieves the largest CAL (2.19 mm) and ABL (2.40 mm) gains, with no increased adverse events; *PRP*: Strong recommendation for patients at high risk of adverse events (e.g., history of bleeding disorders, smokers). Moderate‐quality evidence confirms PRP reduces adverse events by 69%, with a trend toward PD reduction; *Osseous Grafting*: Weak (conditional) recommendation against routine use. Moderate‐quality evidence shows modest CAL gain (0.89 mm, just below the 1 mm clinical threshold) but a 6.87‐fold higher adverse event risk. It may be considered only in patients with large bone defects where GBR is not feasible, with pre‐operative counseling on complication risk; *GTR*: Moderate recommendation for patients with isolated soft tissue defects. Moderate‐quality evidence shows consistent CAL (1.61 mm) and PD (1.22 mm) improvements, with no safety concerns; and (3) Limitations to recommendations: Moderate‐quality evidence means future high‐quality RCTs could change these recommendations and recommendations do not apply to patients with systemic diseases, as these were excluded from included studies—indirectness would downgrade evidence quality for this subgroup.

This study has some limitations. First, although a relatively large number of participants were included, many studies focusing specifically on adverse event severity were excluded, as this was not the primary focus. Second, significant heterogeneity existed among the included studies. Despite subgroup and sensitivity analyses, a comprehensive explanation of heterogeneity sources could not be provided, which may affect the reliability and consistency of the findings. Third, this analysis was based on published literature. The absence of individual participant data introduced potential publication bias and limited the ability to conduct more granular or exploratory analyses, which may have restricted the depth and generalizability of the conclusions. Fourth, we were unable to comprehensively discuss the cost‐effectiveness or accessibility of RPT modalities due to a lack of standardized economic data. None of the included RCTs reported detailed cost information. Fifth, the geographical skew toward Western studies limits the generalizability of our findings to non‐Western global populations. Sixth, several subgroups (e.g., GBR for PD reduction and ABL gain) included only 1 study, making pooled analysis and significance testing methodologically invalid. Seventh, incomplete and heterogeneous data on participants' baseline periodontal status prevent us from determining whether patients with established periodontitis benefit more from RPT. Eighth, incomplete and unstratified data on the adjacent second molar's baseline periodontal status prevent us from determining whether patients with pre‐existing periodontal concerns around this tooth derive greater benefit from RPT. Ninth, the lack of explicit maxillary vs. mandibular third molar specification and stratified outcomes prevents us from analyzing whether RPT efficacy differs by anatomical location. Tenth, the majority of included studies (87.5%) had short‐to‐medium follow‐up times (≤ 6 months), with only 12.5% extending to 9–12 months and none beyond 1 year. This limits our ability to assess the long‐term stability of RPT's effects—critical for periodontal therapy, as late attachment loss or bone resorption can occur 12–24 months posttreatment. Eleventh, moderate heterogeneity in adverse event reporting stems from three key methodological inconsistencies across studies: (1) studies lacked standardized criteria for what constituted an “adverse event”; (2) incomplete severity grading; and (3) many studies reported ≤ 2 adverse events total, leading to unstable within‐study estimates. Twelfth, unmeasured differences in surgical technique between GTR and GBR—coupled with their distinct target sites—introduce potential confounding that may influence our subgroup efficacy findings. Finally, the inclusion of data from unpublished sources or nonpeer‐reviewed platforms, while a strength in mitigating publication bias, may also be considered a limitation. The methodological rigor and data reporting in these sources can be more variable than in formally peer‐reviewed journal articles. Although we applied the same risk‐of‐bias assessment to all included studies, the potential for lower reporting quality in some unpublished records remains.

## 5 Conclusions

Conclusively, this study confirms that RPT significantly enhances tissue regeneration following the extraction of impacted third molars. Compared to conventional treatment, RPT results in an average CAL gain of 1.69 mm, a PD reduction of 1.13 mm, and an ABL gain of 1.31 mm, with no statistically significant difference in the overall risk of adverse events. Although current evidence supports the favorable balance between RPT's efficacy and safety, our novel technique‐specific findings (e.g., superior efficacy of GBR, safety advantages of PRP) highlight the need for future research to: (1) assess long‐term tissue stability (≥ 12 months) of these optimal RPT modalities; (2) investigate biomaterial degradation kinetics tailored to GBR/PRP protocols; and (3) validate our results in larger cohorts with diverse impaction types—all of which stem from the foundational insights of our study.

## Author Contributions

**Xueping Ou:** investigation, writing – original draft, methodology, visualization, formal analysis, data curation. **Shaofei Ma:** conceptualization, investigation, methodology, validation, writing – review and editing, project administration, software, formal analysis, data curation, supervision, resources.

## Disclosure

No AI tools or software were used in the conception, drafting, revision, or finalization of this manuscript. All content was independently created and edited by the authors.

## Ethics Statement

The authors have nothing to report.

## Consent

The authors have nothing to report.

## Conflicts of Interest

The authors declare no conflicts of interest.

## Supporting information


**Data S1:** odi70150‐sup‐0001‐Supinfo.docx.

## Data Availability

The data that support the findings of the study are available from the corresponding authors upon reasonable request.
