# Supplementary material for: Efficacy and Safety of Regenerative Periodontal Therapy on Recovery After Surgical Removal of Impacted Third Molars: A Systematic Review and Meta‐Analysis
Source: Oral Dis. 2025 Dec 15;32(4):966–82. doi: 10.1111/odi.70150 (PMC13248587; doi:10.1111/odi.70150)
Supplement: Supplementary file 1 — Data S1: odi70150‐sup‐0001‐Supinfo.docx. [file ODI-32-966-s001.docx]

**File 1:** Search strategy:

((((“molar, third” [MH] OR third molar* [TIAB] OR wisdom teeth* [TIAB] OR wisdom tooth* [TIAB] OR 3rd molar* [TIAB]))) AND ((“tooth extraction” [MH] OR extraction* [TIAB] OR removal* [TIAB] OR exodontia* [TIAB]))) AND ((“regeneration” [MH] OR “wound healing” [MH] OR “guided tissue regeneration, periodontal” [MH] OR “bone substitutes” [MH] OR guided bone regeneration* [TIAB] OR “autografts” [MH] OR “allografts” [MH] OR xenograft* [TIAB] OR alloplastic* [TIAB] OR “membranes, artificial” [MH] OR membrane* [TIAB] OR barrier* [TIAB] OR growth factor* [TIAB] OR “platelet-derived growth factor” [MH] OR platelet* [TIAB] OR platelet-rich fibrin* [TIAB] OR “platelet-rich plasma” [MH] OR enamel matrix protein derivate* [TIAB] OR emdogain* [TIAB]))

**File 2:** List of Excluded Studies and Reasons for Exclusion and referenced

**Inconsistent disease status**

1. Yuzbasioglu A, Eroglu CN. Evaluating the effectiveness of advanced platelet-rich fibrin, photobiomodulation, pentoxifylline, and Alveogyl in the treatment of alveolar osteitis: a randomized controlled clinical trial. BMC Oral Health. 2024 Dec 26;24(1):1559. doi: 10.1186/s12903-024-05372-6.
2. Leung YY. Guided bone regeneration to reduce root migration after coronectomy of lower third molar: a randomized clinical trial. Clin Oral Investig. 2019 Apr;23(4):1595-1604. doi: 10.1007/s00784-018-2594-8.
3. Aravena PC, Sandoval SP, Pizarro FE, Simpson MI, Castro-Adams N, Serandour G, Rosas C. Leukocyte and Platelet-Rich Fibrin Have Same Effect as Blood Clot in the 3-Dimensional Alveolar Ridge Preservation. A Split-Mouth Randomized Clinical Trial. J Oral Maxillofac Surg. 2021 Mar;79(3):575-584. doi: 10.1016/j.joms.2020.10.006.
4. Pereira DA, Mendes PGJ, Prisinoto NR, de Rezende Barbosa GL, Soares PBF, de Oliveira GJPL. Advanced platelet-rich-fibrin (A-PRF +) has no additional effect on the healing of post-extraction sockets of upper third molars. A split mouth randomized clinical trial. Oral Maxillofac Surg. 2023 Sep;27(3):411-419. doi: 10.1007/s10006-022-01075-w.
5. Elayah SA, Younis H, Cui H, Liang X, Sakran KA, Alkadasi B, Al-Moraissi EA, Albadani M, Al-Okad W, Tu J, Na S. Alveolar ridge preservation in post-extraction sockets using concentrated growth factors: a split-mouth, randomized, controlled clinical trial. Front Endocrinol (Lausanne). 2023 May 17;14:1163696. doi: 10.3389/fendo.2023.1163696.
6. Uribe-Fentanes LK, Soriano-Padilla F, Pérez-Frutos JR, Veras-Hernández MA. Acción del extracto de Calendula officinalis en la preservación ósea posterior a extracción [Action of Calendula officinalis essence on bone preservation after the extraction]. Rev Med Inst Mex Seguro Soc. 2018 Jan-Feb;56(1):98-105. Spanish.
7. Varghese MP, Manuel S, Kumar L K S. Potential for Osseous Regeneration of Platelet-Rich Fibrin-A Comparative Study in Mandibular Third Molar Impaction Sockets. J Oral Maxillofac Surg. 2017 Jul;75(7):1322-1329. doi: 10.1016/j.joms.2017.01.035.
8. McNamara Z, Findlay G, O'Rourke P, Batstone M. Removal versus retention of asymptomatic third molars in mandibular angle fractures: a randomized controlled trial. Int J Oral Maxillofac Surg. 2016 May;45(5):571-4. doi: 10.1016/j.ijom.2016.01.007.
9. Leung YY. Coronectomy of lower third molars with and without guided bony regeneration: a pilot study. Br J Oral Maxillofac Surg. 2016 Feb;54(2):155-9. doi: 10.1016/j.bjoms.2015.12.010.
10. Yolcu Ü, Acar AH. Comparison of a new flap design with the routinely used triangular flap design in third molar surgery. Int J Oral Maxillofac Surg. 2015 Nov;44(11):1390-7. doi: 10.1016/j.ijom.2015.07.007.
11. Baslarli O, Tumer C, Ugur O, Vatankulu B. Evaluation of osteoblastic activity in extraction sockets treated with platelet-rich fibrin. Med Oral Patol Oral Cir Bucal. 2015 Jan 1;20(1):e111-6. doi: 10.4317/medoral.19999.
12. Aydintug YS, Bayar GR, Gulses A, Misir AF, Ogretir O, Dogan N, Sencimen M, Acikel CH. Clinical study on the closure of extraction wounds of partially soft tissue-impacted mandibular third molars. Quintessence Int. 2012 Nov-Dec;43(10):863-70.
13. Gürbüzer B, Pikdöken L, Tunali M, Urhan M, Küçükodaci Z, Ercan F. Scintigraphic evaluation of osteoblastic activity in extraction sockets treated with platelet-rich fibrin. J Oral Maxillofac Surg. 2010 May;68(5):980-9. doi: 10.1016/j.joms.2009.09.092
14. Gürbüzer B, Pikdöken L, Urhan M, Süer BT, Narin Y. Scintigraphic evaluation of early osteoblastic activity in extraction sockets treated with platelet-rich plasma. J Oral Maxillofac Surg. 2008 Dec;66(12):2454-60. doi: 10.1016/j.joms.2008.03.006.
15. Bauss O, Schwestka-Polly R, Kiliaridis S. Influence of orthodontic derotation and extrusion on pulpal and periodontal condition of autotransplanted immature third molars. Am J Orthod Dentofacial Orthop. 2004 Apr;125(4):488-96. doi: 10.1016/j.ajodo.2003.11.018.

**Absence of an effective control group**

1. Laiamnuay P, Chaiyasamut T, Zhang R, Seriwatanachai D, Kretapirom K. Comparable efficacy of autogenous tooth-derived grafts and xenografts in post-extraction osseous defects: a split-mouth randomized trial. Clin Oral Investig. 2025 Jul 23;29(8):388. doi: 10.1007/s00784-025-06458-3.
2. Alves Pereira D, Silva Bonatto M, Souza Santos S, Gomes Junqueira Mendes P, Sales E Pessoa R, Pimentel Lopes de Oliveira GJ. Comparison of the effects of antibiotic therapy and photobiomodulation with red and infrared lasers on the healing of postextraction sockets of third molars: A randomised controlled trial. Lasers Med Sci. 2025 Apr 17;40(1):196. doi: 10.1007/s10103-025-04445-4.
3. Dudak ME, Calis A, Koca H. Effectiveness of Concentrated Growth Factor on Improving Postoperative Comfort in Patients Undergoing Coronectomy for Impacted Lower Third Molars: A Double-Blinded Split-Mouth Randomized Controlled Clinical Study. J Oral Maxillofac Surg. 2025 Jun;83(6):738-747. doi: 10.1016/j.joms.2025.03.005.
4. He X, Gao Y, Shen J, Pan Q, Chen J, He Y. Removal of Horizontally Impacted Mandibular Third Molars Using the Three-Piece or T-Shaped Tooth Sectioning Method. J Oral Maxillofac Surg. 2024 Aug;82(8):968-975. doi: 10.1016/j.joms.2024.03.036.
5. Pereira DA, Bonatto MS, Santos SS, Mendes PGJ, Sales E Pessoa R, de Oliveira GJPL. Comparison of different dual-wavelength photobiomodulation protocols application in third molar extractions. A split-mouth randomized controlled trial. Photodiagnosis Photodyn Ther. 2024 Apr;46:104054. doi: 10.1016/j.pdpdt.2024.104054.
6. Rodrigues ED, Pontual AD, Macedo RA, Nascimento E, Vasconcelos BC. Evaluation of bone repair with platelet-rich fibrin following the extraction of impacted third molars - randomized clinical trial. Med Oral Patol Oral Cir Bucal. 2023 Sep 1;28(5):e433-e441. doi: 10.4317/medoral.25856.
7. Sáez-Alcaide LM, Molinero-Mourelle P, González-Serrano J, Rubio-Alonso L, Bornstein MM, López-Quiles J. Efficacy of a topical gel containing chitosan, chlorhexidine, allantoin and dexpanthenol for pain and inflammation control after third molar surgery: A randomized and placebo-controlled clinical trial. Med Oral Patol Oral Cir Bucal. 2020 Sep 1;25(5):e644-e651. doi: 10.4317/medoral.23661.
8. Pachipulusu PK, S M. Comparative study of primary and secondary closure of the surgical wound after removal of impacted mandibular third molars. Oral Maxillofac Surg. 2018 Sep;22(3):261-266. doi: 10.1007/s10006-018-0696-8.

**Insufficient data**

1. Pereira DA, Bonatto MS, Soares EC Jr, Mendes PGJ, Pessoa RSE, de Oliveira GJPL. Photobiomodulation With Infrared and Dual-Wavelength Laser Induces Similar Repair and Control of Inflammation After Third Molar Extraction: A Double-Blinded Split-Mouth Randomized Controlled Trial. J Oral Maxillofac Surg. 2025 Mar;83(3):332-343. doi: 10.1016/j.joms.2024.11.009.
2. Erdem MK, Cambazoglu M. A comparative analysis of postoperative morbidity and alveolar bone regeneration following surgical extraction of ımpacted lower third molar teeth using piezosurgery and conventional ınstruments: a split-mouth clinical ınvestigation. Eur J Med Res. 2024 Sep 14;29(1):460. doi: 10.1186/s40001-024-02051-8.
3. Demirok SO, Eroglu CN, Koc A. Comprehensive analysis of bone tissue in extraction sockets of third molars after leukocyte and platelet rich fibrin and photobiomodulation applications. Clin Oral Investig. 2024 Aug 13;28(9):483. doi: 10.1007/s00784-024-05872-3.
4. Huang C, Xu Y. Can concentrated growth factor prevent postoperative complications of impacted third molar surgery? A split-mouth randomized double-blind trial. Clin Oral Investig. 2024 Apr 1;28(4):234. doi: 10.1007/s00784-024-05638-x.
5. Rodrigues ÉDR, Martins-de-Barros AV, Loureiro AMLC, Carvalho MV, Vasconcelos B. Comparison of two suture techniques on the inflammatory signs after third molars extraction-A randomized clinical trial. PLoS One. 2023 Jun 23;18(6):e0286413. doi: 10.1371/journal.pone.0286413.
6. Afat IM, Akdoğan ET, Gönül O. Effects of leukocyte- and platelet-rich fibrin alone and combined with hyaluronic acid on early soft tissue healing after surgical extraction of impacted mandibular third molars: A prospective clinical study. J Craniomaxillofac Surg. 2019 Feb;47(2):280-286. doi: 10.1016/j.jcms.2018.11.023.

**Review articles**

1. Salas GA, Lai SA, Verdugo-Paiva F, Requena RA. Platelet-Rich Fibrin in Third Molar Surgery: Systematic Review and Meta-Analysis Protocol. Craniomaxillofac Trauma Reconstr. 2022 Jun;15(2):164-168. doi: 10.1177/19433875211016203.
2. Domic D, Bertl K, Lang T, Pandis N, Ulm C, Stavropoulos A. Hyaluronic acid in tooth extraction: a systematic review and meta-analysis of preclinical and clinical trials. Clin Oral Investig. 2023 Dec;27(12):7209-7229. doi: 10.1007/s00784-023-05227-4.
3. Siawasch SAM, Yu J, Castro AB, Temmerman A, Teughels W, Quirynen M. Autologous platelet concentrates after third molar extraction: A systematic review. Periodontol 2000. 2025 Feb;97(1):131-152. doi: 10.1111/prd.12600.
4. Pattnayak A, Ramanna PK, Mahabala KY, Edathotty TT, Kumaraswamy AU, Duseja S. Impact of Platelet-rich Plasma and Platelet-rich Fibrin in Mandibular Third Molar Extraction: A Systematic Review. J Contemp Dent Pract. 2024 Sep 1;25(9):904-910. doi: 10.5005/jp-journals-10024-3727.
5. Raghavan SL, Sivakumar G, Sivakumar S. Effectiveness of Reso-Pac in enhancing wound healing after third molar surgery: a systematic review with meta-analysis of randomized controlled trials. BMC Oral Health. 2025 Aug 23;25(1):1369. doi: 10.1186/s12903-025-06459-4.

**File 3:**


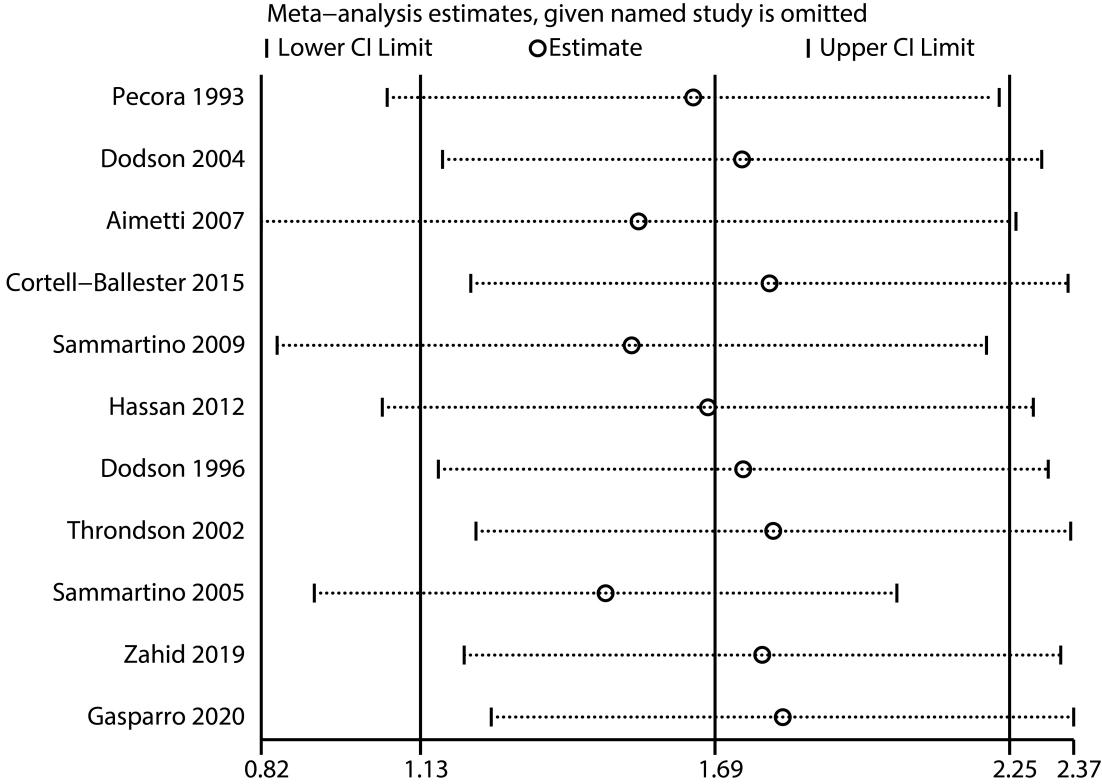


Figure S1. Sensitivity analysis for the effect of regenerative periodontal therapy on CAL gain


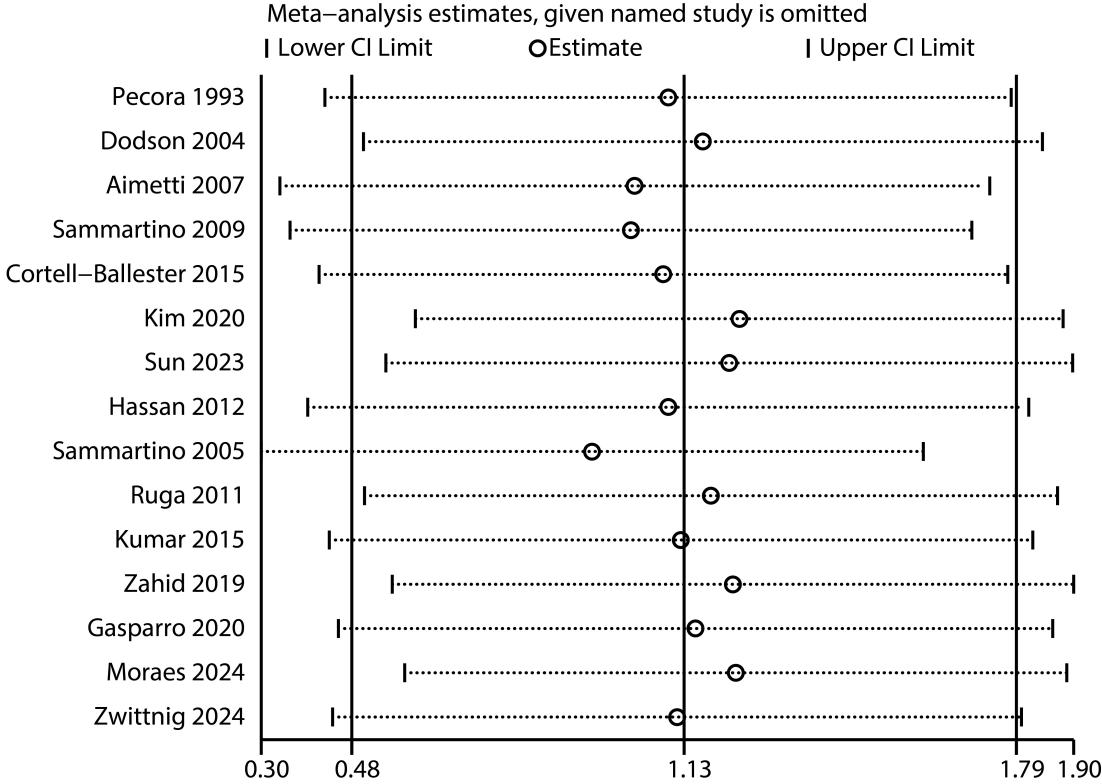


Figure S2. Sensitivity analysis for the effect of regenerative periodontal therapy on PD reduction


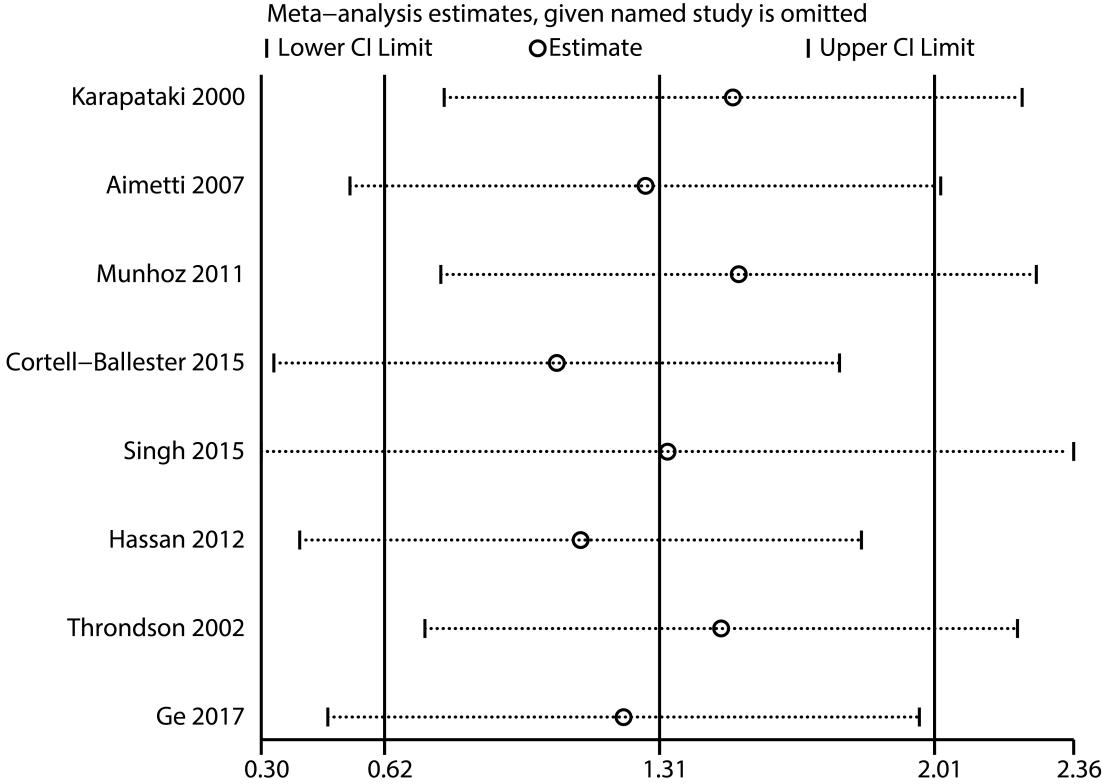


Figure S3. Sensitivity analysis for the effect of regenerative periodontal therapy on ABL gain.


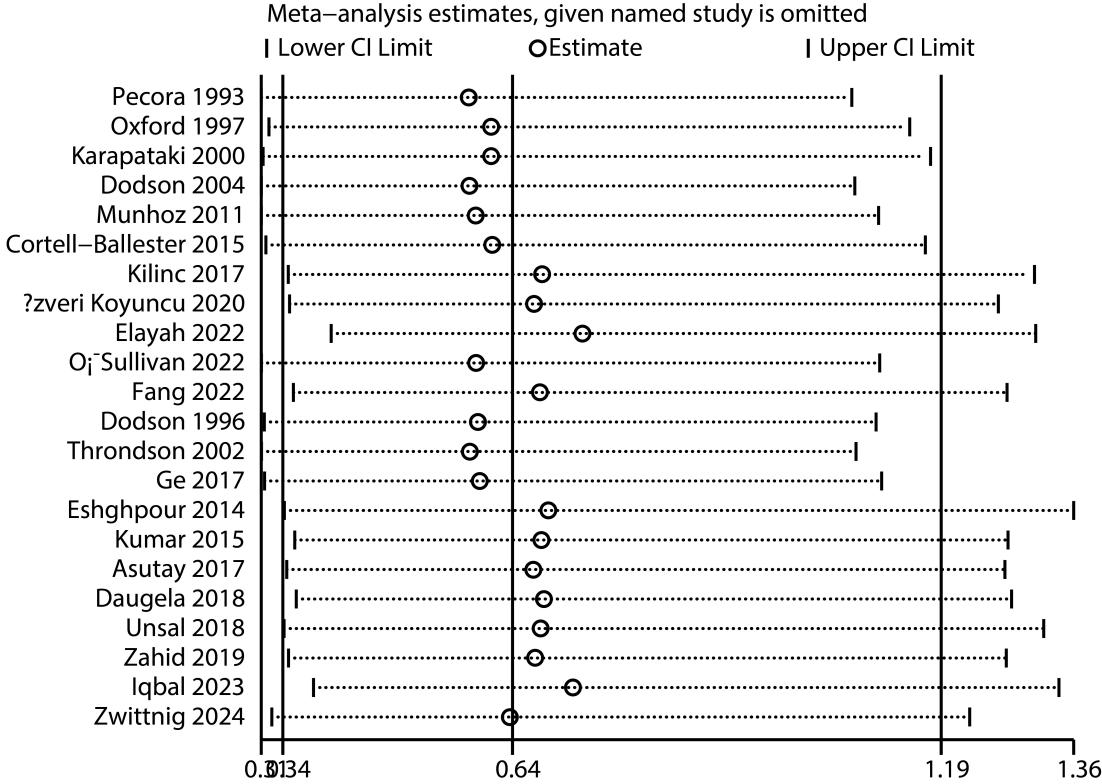


Figure S4. Sensitivity analysis for the effect of regenerative periodontal therapy on the risk of adverse events.

**File 4:**


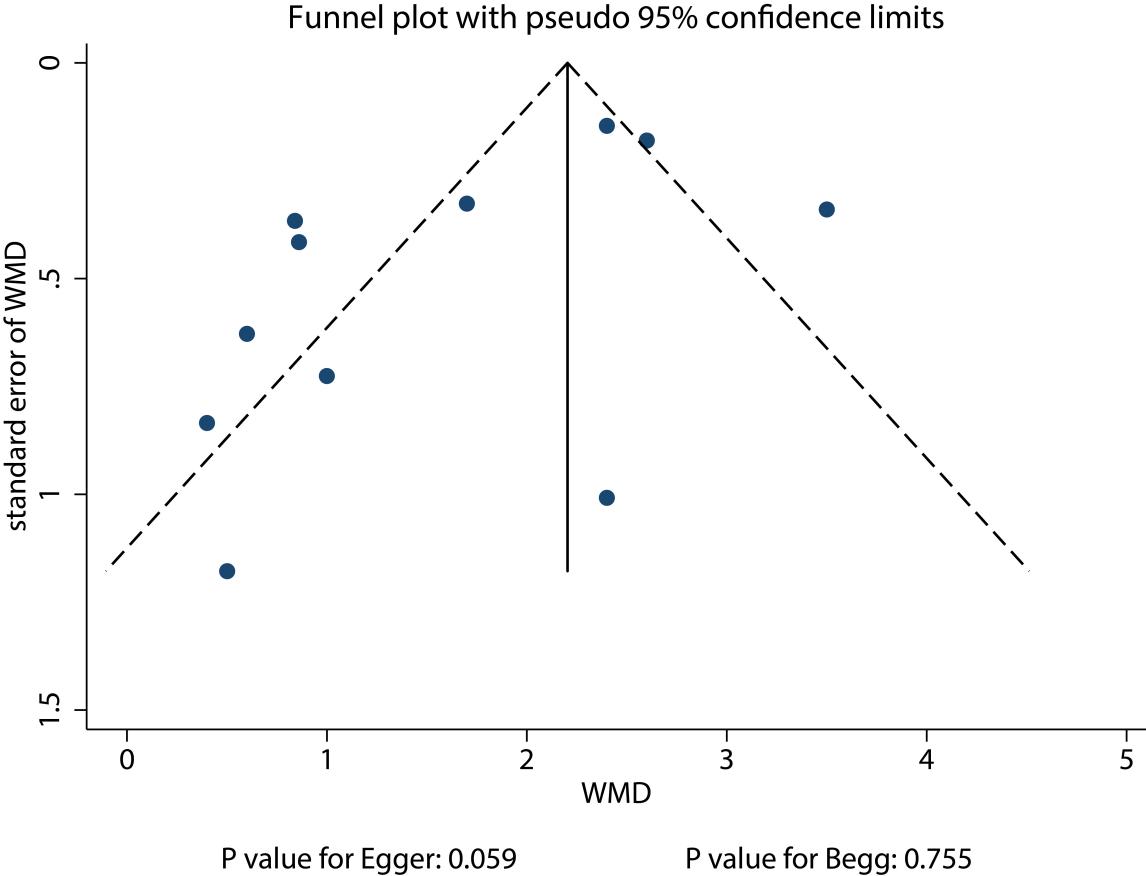


Figure S1. Funnel plot for CAL gain


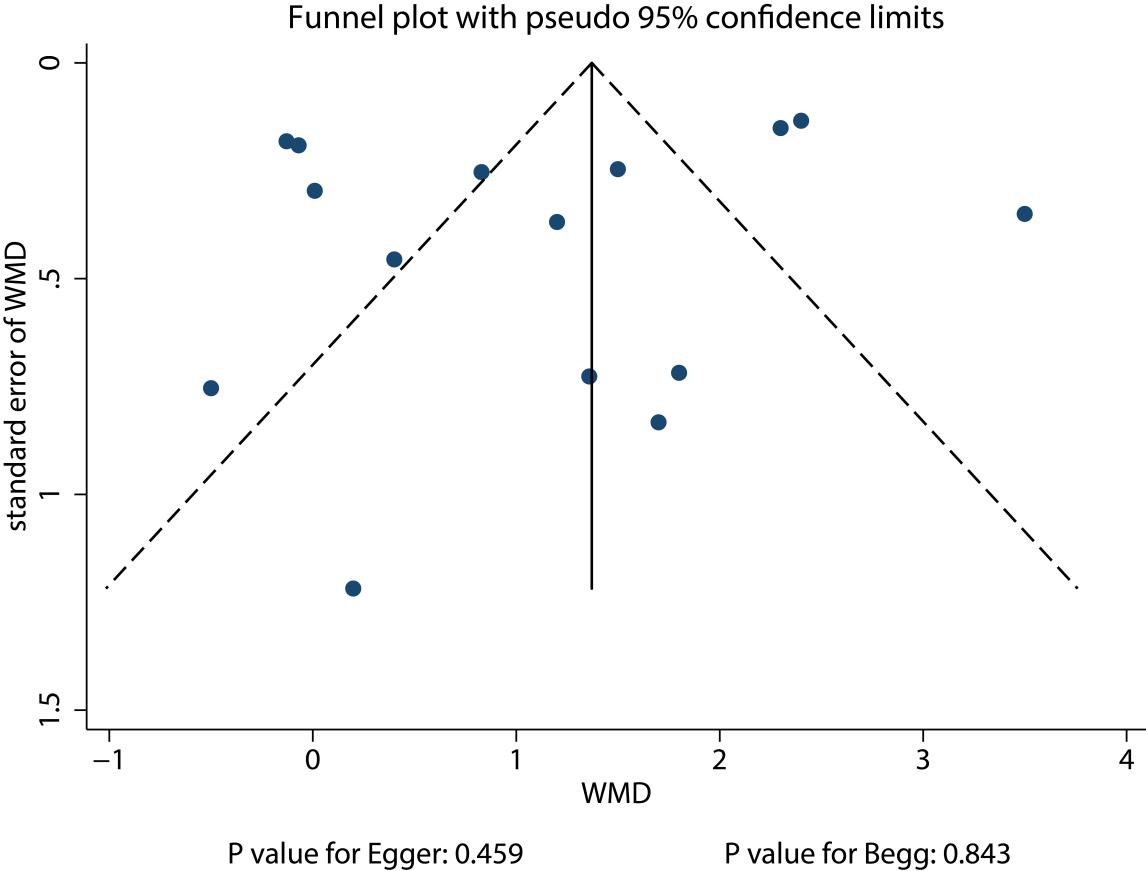


Figure S2. Funnel plot for PD reduction


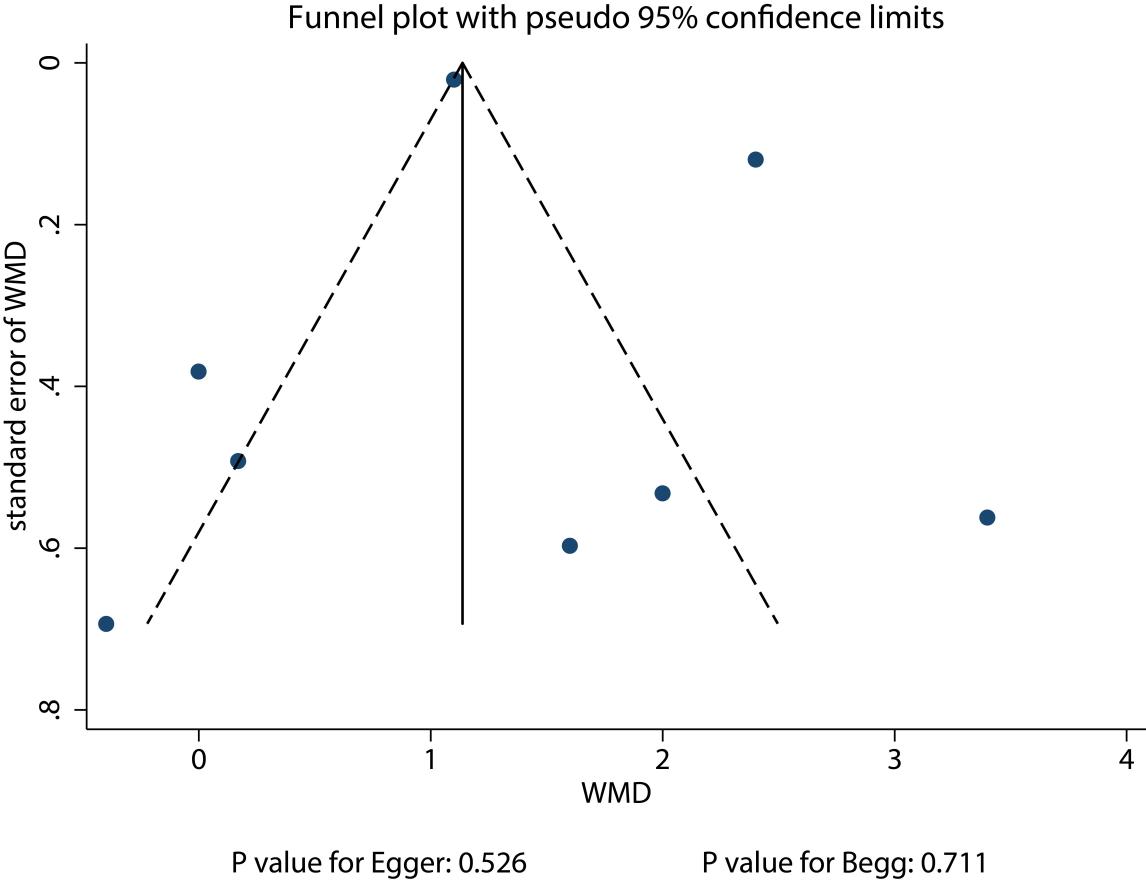


Figure S3. Funnel plot for ABL gain


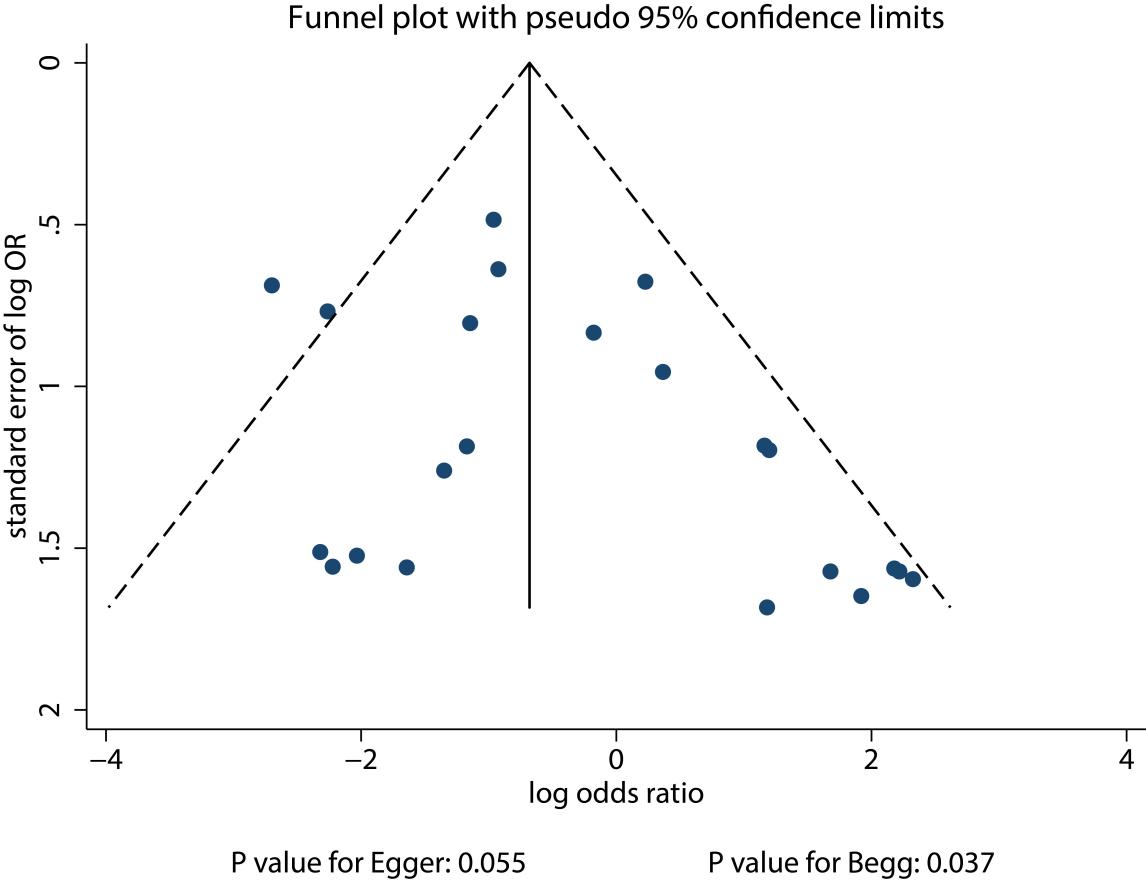


Figure S4. Funnel plot for adverse events
